# The Mitochondrial Genome of Soybean Reveals Complex Genome Structures and Gene Evolution at Intercellular and Phylogenetic Levels

**DOI:** 10.1371/journal.pone.0056502

**Published:** 2013-02-19

**Authors:** Shengxin Chang, Yankun Wang, Jiangjie Lu, Junyi Gai, Jijie Li, Pu Chu, Rongzhan Guan, Tuanjie Zhao

**Affiliations:** 1 National Center for Soybean Improvement, Nanjing, Jiangsu, China; 2 Key Laboratory of Biology and Genetic Improvement of Soybean, Ministry of Agriculture, Nanjing, Jiangsu, China; 3 National Key Laboratory for Crop Genetics and Germplasm Enhancement, Nanjing Agricultural University, Nanjing, Jiangsu, China; University of Georgia, United States of America

## Abstract

Determining mitochondrial genomes is important for elucidating vital activities of seed plants. Mitochondrial genomes are specific to each plant species because of their variable size, complex structures and patterns of gene losses and gains during evolution. This complexity has made research on the soybean mitochondrial genome difficult compared with its nuclear and chloroplast genomes. The present study helps to solve a 30-year mystery regarding the most complex mitochondrial genome structure, showing that pairwise rearrangements among the many large repeats may produce an enriched molecular pool of 760 circles in seed plants. The soybean mitochondrial genome harbors 58 genes of known function in addition to 52 predicted open reading frames of unknown function. The genome contains sequences of multiple identifiable origins, including 6.8 kb and 7.1 kb DNA fragments that have been transferred from the nuclear and chloroplast genomes, respectively, and some horizontal DNA transfers. The soybean mitochondrial genome has lost 16 genes, including nine protein-coding genes and seven tRNA genes; however, it has acquired five chloroplast-derived genes during evolution. Four tRNA genes, common among the three genomes, are derived from the chloroplast. Sizeable DNA transfers to the nucleus, with pericentromeric regions as hotspots, are observed, including DNA transfers of 125.0 kb and 151.6 kb identified unambiguously from the soybean mitochondrial and chloroplast genomes, respectively. The soybean nuclear genome has acquired five genes from its mitochondrial genome. These results provide biological insights into the mitochondrial genome of seed plants, and are especially helpful for deciphering vital activities in soybean.

## Introduction

The size of the mitochondrial genome (mtDNA) in seed plants is highly variable [1], [2], ranging from 208 kb in white mustard [3] to 11.3 Mb in *Silene conica* [4]. However, even the smallest plant mitochondrial genome is much larger than that of animals (typically ∼ 16 kb) [5]. Mitochondrial genomes in seed plants are enriched with repeats, such as tandem repeats, short repeats and large repeats [6]–[8]. The short repeats mediate irreversible recombination of the mitochondrial genome, which results in inheritable changes to the genome structure [9], [10]. The large repeats, usually larger than 1 kb, may mediate reversible recombination of the mitochondrial genomes, regulate the molecular conformation of the genome, and may reflect or predict the possible constituents of the genome molecular pools that function in energy metabolism of plants [11]. The largest number of pairwise large repeats (nine pairs) was reported in a wheat K-type cytoplasmic male sterility (CMS) line with a moderate mitochondrial genome size [12]. The smallest mitochondrial genome (white mustard) does not have any large repeats [3].

The mitochondrial genome size, number of pairwise large repeats and structural organization vary with mitotypes specific to a plant species. The profiles of genes of known function that are necessary for energy generation also differ significantly because of loss and acquisition of genes during the evolution of higher plants [6]. Predicted open reading frames (ORFs) of undefined function in mitochondrial genomes vary more than the genes of known function. The predicted ORFs, which vary significantly among plant species within a closely related clade [13], are usually expressed in plants, and thus have some function in the activities of plants [6], [13]. However, except for several ORFs related to CMS, these ORFs have not been extensively studied.

Coexistent mitotypes, which include major and minor mitotypes [14]–[16], are recent discoveries in seed plants. The minor mitotype is usually difficult to detect, is present in several fold fewer copies than the major mitotype, and has no obvious function in the mitochondria. However, the minor mitotype may change into the major mitotype under certain conditions related to male fertility change [17].

Limited studies have shown that mitochondrial genomes of soybean (*Glycine max*) are more complex than many others. Electron microscopy showed that the genome has seven apparent size classes with unequal mean lengths [18]. Soybean mitochondrial genomes were classified into four types by restriction fragment length polymorphism analysis [19] and a large repeat of 4.8 kb has been detected [20]. The soybean mitochondrial genome length was estimated to be about 392 kb [21]. Recently, three mitochondrial genomes in the legume family (*Vigna radiata*, *Lotus japonicus* and *Millettia pinnata*) have been characterized [7]. These genomes, with rather simple structures relative to soybean, may act as references for the soybean mitochondrial genome.

Soybean is one of the most important global crops, grown for vegetable oil and protein. In 2010–2011, its production comprised 58.0%, 67.9% and 28.0% of the world’s major oilseeds production, major protein meals and vegetable oils production, respectively (http://www.fas.usda.gov/oilseeds/Current/). Soybean nuclear and chloroplast genomes have been published [22], [23], greatly increasing our understanding of soybean biology. Although biologists have been investigating the soybean mitochondrial genome for more than 30 years, its sequences remain obscure because of its complex structures. High-throughput sequencing technology provides an efficient method to study the soybean mitochondrial genome. In the present work, high throughput sequencing was used to obtain the complete sequence of the mitochondrial genome of a popular soybean variety planted in the Huang-Huai river valley of China where soybean originated. The sequence revealed that the structure of the soybean mitochondrial genome is the most complex among seed plant genomes sequenced so far. Transfers of DNA among the nuclear, mitochondrial and chloroplast genomes in soybean were also revealed. Gene losses and acquisitions were identified after constructing a phylogenetic tree with mitochondrial genomes of 28 representative species of higher plants. The data presented here represent a significant advance in plant biology, especially in soybean research.

## Results

### Mitochondrial Genome Assembly

Soybean mtDNA was sequenced by a Roche 454 GS FLX. 15,861 reads covering 6,317,283 bp were generated, and the data were assembled into 33 contigs. Searching with these contigs against the reported nuclear and chloroplast genomes of soybean [22], [23] showed that five of the contigs (<500 bp) that had low sequencing coverage depths of 3–4 are contaminant DNAs: three are nuclear genome sequences and two are chloroplast genome (cpDNA) sequences. These sequences were removed from the assembly of the soybean mitochondrial genome.

The longest of the 28 contigs was 73,279 bp. The contig length at the 50% quantile position was 31,090 bp (N50 value, defined in the Newbler software). The maximum contig length and N50 value are both very large, indicating that the sequencing data are good enough to assemble the genome. The bb.454contignet software was used to join the contigs and obtain a connecting map. To verify the 36 connections of contigs, we designed corresponding primers (Table S1 in File S1) and the expected bands were obtained for all 36 primer sets (Figure S1). Sanger sequencing was then applied to verify the assembly.

These assembly results showed that 14 of the contigs were assembled into the mitochondrial genome once, and the other 14 contigs were linked twice or more on the contigs map. These results indicated that certain sequences have multiple copies in the genome.

Using contig 3 as the start point of the assembly, we linked the contigs sequentially on the connecting map (Figure S2). A genome covering all of the contigs was then generated with a complete length of 402.5 kb, which is the single master circle of the genome. Furthermore, we analyzed the coverage depths of the repeat regions in this genome and discovered that the putative repeated sequences had much higher coverage depths than the single copy sequences, which further demonstrated the existence of the repeats (Figure S3).

The resultant circular master genome covers all of the genome information. This assembly is not the sole complete soybean genome because of the presence of isometric genome structures that will be detailed in a later section.

### Gene Content of the Soybean Genome

Sequencing the mitochondrial genome of *G. max* produced a complete molecule of 402,558 bp (Genbank accession number: JX463295), which is larger than *L*. *japonicus* (380.9 kb) and *V. radiata* (401.3 kb) [7], but smaller than *M. pinnata* (425.7 kb) among legumes. Its G+C content is 45.0%, comparable to other sequenced plant mitochondrial genomes (maize, 43.9% [24]; rice, 43.8% [25]; sugar beet, 43.9% [26]; *Arabidopsis thaliana*, 44.8% [27]; and *Brassica* mitotypes, about 45% [13], [28]).

**Figure 1:**
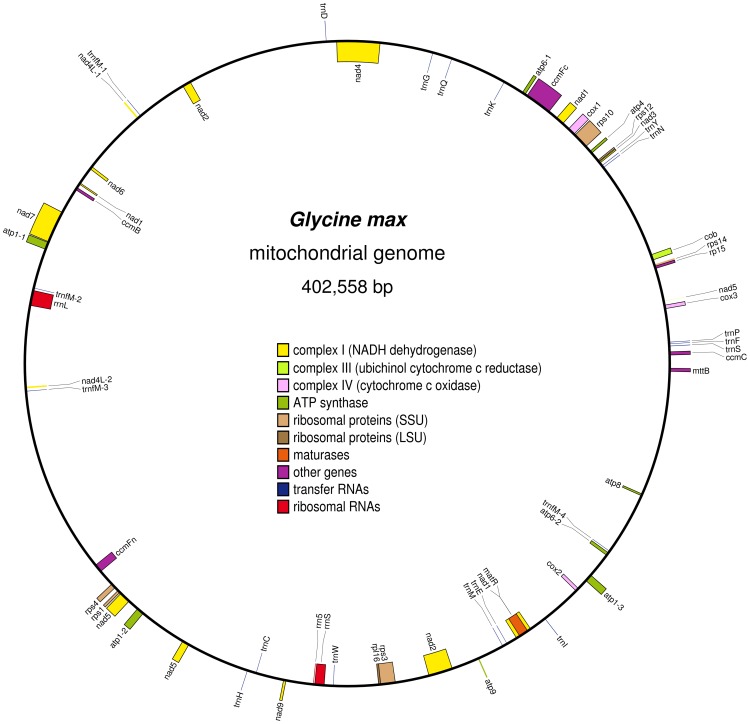
The circular map of the mitochondrial genome of *G*. *max*. Features on the clockwise- and counter-clockwise-transcribed strands are drawn on the inside and outside of the circle, respectively. The figure was drawn using OGDraw v1.2 [65].

Using BLAST and tRNA scan-SE, 58 genes (73,389 bp in total length) were identified, including 36 protein-coding genes, three rRNA genes (5S, 18S and 26S rRNAs) and 19 tRNA genes. Genes of known function account for 18.2% of the whole genome (Table S2 in File S1). The positions of these genes in the soybean mitochondrial genome are shown in Figure 1. Among genes of known function, *nad4L* and *atp6* are present in two copies, *atp1* has three copies, there are three copies of the fifth exon of *nad7*, and *trnfM*-CAT has four copies. In comparison with other legume mitochondrial genomes, the *G. max* genome has the same types of genes as *M. pinnata*, but is slightly different from that of *V. radiata* and *L. japonicus* (Figure S4). Additionally, the soybean mitochondrial genome is predicted to have 52 ORFs of unknown function.

### Repeats

The repeats in the soybean mtDNA include short, tandem and large repeats, and total 76.4 kb, accounting for 19.0% of the genome. The short repeats represent less than 1 kb, and are mainly less than 500 bp (Table S3 in File S1), similar to other plant mitochondrial genomes [8], [13]. Short repeats account for 4.2% of the genome, and are uniformly distributed in the genome, as demonstrated by the Kolmogorov–Smirnov test. Short repeats may be used to analyze the structural reorganization of mitochondrial genomes [7], [25], [26]. Nevertheless, the three mitochondrial genome sequences in the legume family are too evolutionarily distant from the *Glycine* genus to uncover the genomic re-organization processes. Only four tandem repeats (12 bp to 18 bp) were identified in the genome (Table S4 in File S1). The soybean genome has the fewest tandem repeats among the reported mitochondrial genomes of seed plants (Table S5 in File S1).

**Figure 2:**
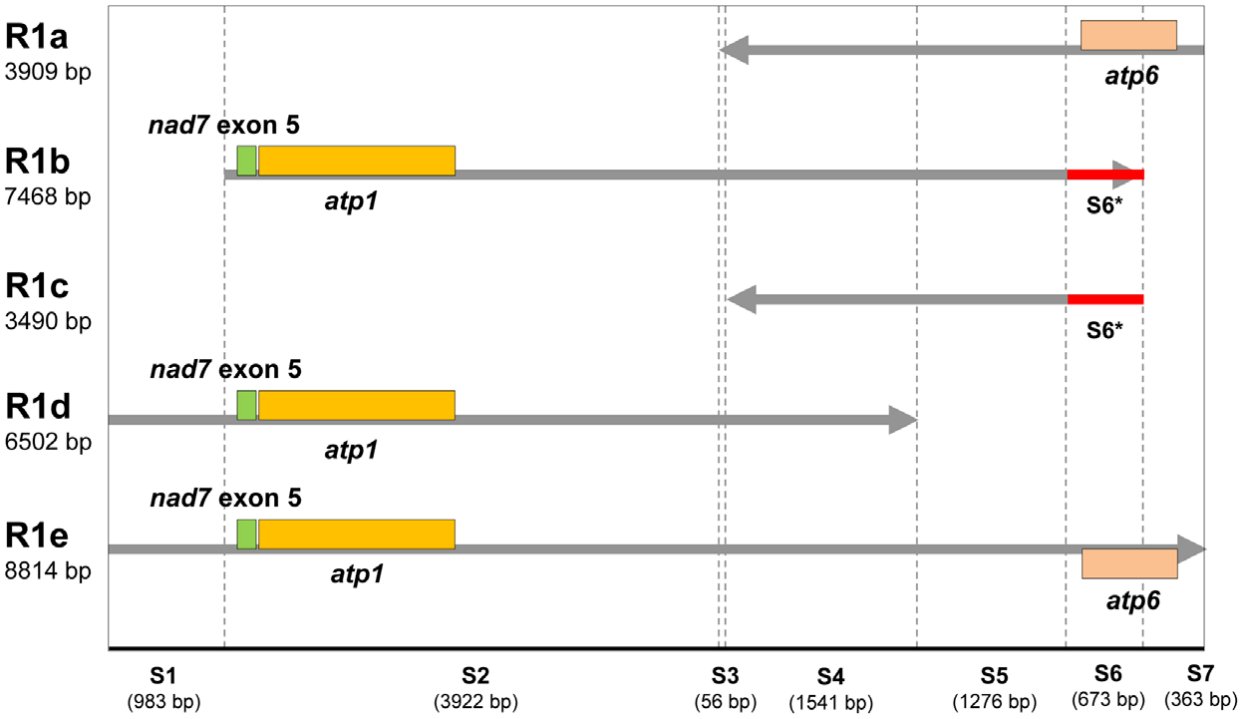
Relationships revealed by multiple alignments among five R1 large repeats (marked as R1a–e). R1b, R1d, R1e are in the some orientation; R1a and R1c are in the reverse orientation. The red segments S6* in R1b and R1c are homologous, but different from S6 in the other three R1 large repeats.

Large repeats (>1 kb) are notable because they are related to reversible genomic structural changes. The soybean mitochondrial genome has four groups of large repeats representing 59,273 bp, and accounting for 14.7% of the genome. The number of pairwise large repeats is 13, which is the highest number among reported mitochondrial genomes of higher plants. We designated the large repeats as R1 (Table 1) and R2–R4 (Table 2). R2 is 4,962 bp in length and has a pair of large repeats in the same orientation. R3 (6,161 bp) and R4 (3,682 bp) have a pair of large repeats in the opposite orientation. R1 has five homologous large repeats of unequal lengths. As shown in Figure 2, the five R1 repeats (designated as R1a–e) have partial segments matched in the alignment with R1e. According to the nearly identical sequences, we partitioned R1e into seven segments, marked as S1–S7 (see Table 1 and Figure 2), the length of which are 56 to 3922 bp. Different R1 repeats carry different linked segments marked with an S. All of the homologous S segments have identities of more than 99%. It is hypothesized that R1a–d evolved from the large progenitor sequence R1e through elimination of partial segments at the ends of R1e, followed by genome recombination during evolution. This resulted in R1a, R1b, R1c and R1d containing two to five linked segments from R1e. R1b and R1c contain the segment S6* (673 bp), marked in red, which is different from the S6 segment in R1a, R1d and R1e. The S6* segments at one end of R1b may have resulted from a recombination event in which the segment shortened from R1e was recombined with sequence containing S6*. R1c might have evolved from R1b through elimination of its partial segments at the end containing the *atp1* gene, which was integrated in the soybean genome at another location.

**Table 1:** Locations of large repeats R1 and homologous sequences in the soybean mitochondrial genome.

Name	Begin	End	Length	Takengenes	Segmentsincluded
R1a	62,957	66,865	3,909	*atp6*	S3, S4, S5, S6, S7
R1b	176,196	183,663	7,468	*atp1*, *nad7*exon 5	S2, S3, S4, S5, S6*
R1c	234,626	238,115	3,490	–	S4, S5, S6*
R1d	255,146	261,647	6,502	*atp1*, *nad7*exon 5	S1, S2, S3, S4
R1e	353,867	362,680	8,814	*atp1*, *atp6*,*nad7* exon 5	S1–S7

S denotes the segments shown in Figure 2. Segment S6* shown in red in Figure 2 is different from segment S6.

**Table 2:** Location of the large repeats (R2–R4) in the soybean mitochondrial genome.

Name	Begin	End	Length	Begin	End	Length	Identity	Orientation	Harboring gene
R2	33,155	37,846	4,692	276,715	281,406	4,692	99%	forward	–
R3	140,148	146,308	6,161	205,656	211,816	6,161	99%	reverse	*nad4L*, *trnfM*
R4	241,273	244,964	3,692	393,772	397,463	3,692	100%	reverse	–

The evolutionary sequence, as shown in Figure 2, could not have been formed through random recombination, thus we speculated that the evolutionary direction of large repeats was from large segments to small segments. Moreover, the large repeats harboring important genes such as *atp1, atp6* and *nad7* exon 5, increased the copy numbers of these genes. These increased numbers of genes involved in energy metabolism in mitochondria probably have important effects in soybean. On the other hand, the increase in copy numbers of these important genes does not equal the increase in copy numbers of the repeats most likely because of the suggested elimination. This may be related to the energy balance of the metabolic system or functional implications of having the appropriate copy number.

### Subgenomic Circles

Plant mitochondrial genomes can be reversibly reorganized to form subgenomic circles through homologous recombination based on the large repeats. The large number of large repeats found in soybean results in a circular molecular pool that is the most complex in reported mitochondrial genomes of seed plants.

**Figure 3:**
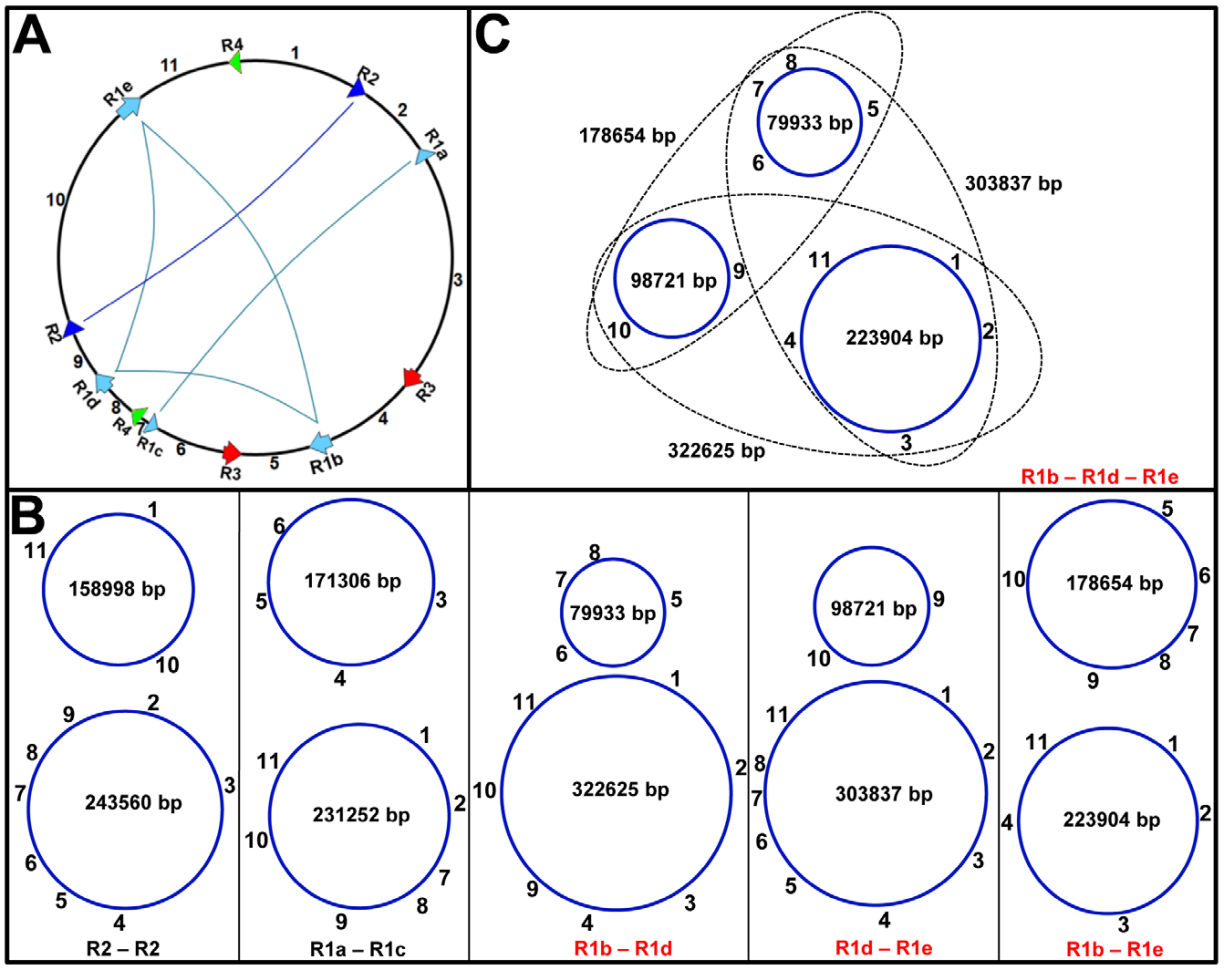
Reversible reorganization of the soybean mtDNA may produce multiple subgenomic circles mediated by large repeats. (A) Arrows of the same color denote homologous large repeats and their sequence orientation. (B) shows subgenomic circles of various sizes produced by rearrangements of the five pairs of large repeats. (C) The three small circles may be produced by three pairs of large repeats (R1b-R1d, R1d-R1e and R1b-R1e).

Pairwise large repeats in the same orientation may produce two small subgenomic circles [7], [12]. For example, the soybean genome in Figure 3A (the same as Figure 1) may produce two subgenomic circles comprising a large subgenomic circle of 322,625 bp and a small subgenomic circle of 79,933 bp, mediated by the pairwise large repeats R1b and R1d (Figure 3B). The large subgenomic circle may further produce two smaller circles of 98,721 bp and 223,904 bp, mediated by another pair of large repeats (R1d and R1e) that are present in this large subgenomic circle (Figure 3C). Joint analysis indicated that R1b, R1d and R1e may produce three small subgenomic circles by three alternative methods, as shown in Figure 3C. When single pairwise large repeats are considered, the mitochondrial genome master circle may produce 10 subgenomic circles (Figure 3B), mediated by various pairwise large repeats in Figure 3A. These subgenomic circles are all validated by the linking pathways of contigs on the connecting map. For example, the circle of 79,933 bp shown in Figure 3C is formed by linking the contigs in order: R1b-7-24- 13-6-23-20-19-26-25-18-17-11-22, while R1b is formed by linking the contigs as 16-28-26-19-20-23 (Figure S2). The circle of 98,721 bp shown in Figure 3C is formed by the contigs linked as R1d-9-15-10-26-25-2, while R1d is formed by contigs linked as 22-16-28-26-19.

**Figure 4:**
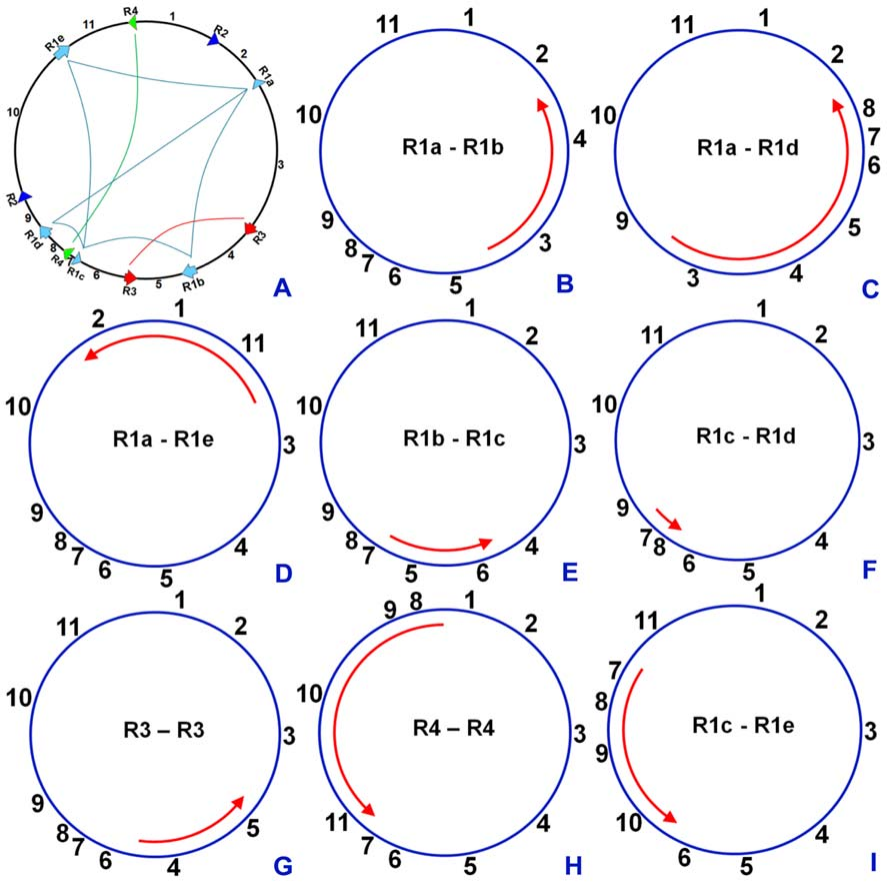
Isometric genome structures formed by rearrangements within eight pairs of inverted large repeats. (A) is structurally the same as Figure 3A. (B–I) shows the eight isometric master genome structures of (A). The red arrows in the circles denote the inverted regions mediated by the repeats. Combinations of inverted repeats for each isometric master circle are marked in the circles.

The pairs of large repeats in the reverse direction may mediate the formation of isometric mitochondrial genome circles equivalent to the master circle shown in Figure 1 and 3A [7], [12]. The sequence obtained has eight pairs of reversed large repeats; therefore, the genome has eight isometric master circular genomic structures (Figure 4B–I). The connecting map provides direct evidence for the existence of these isometric structures (Figure S2). In fact, the genome assembly can also generate these isometric structures. All of the isometric master circles (Figure 4) may produce multiple subgenomic subcircles. This significantly increases the number of possible small circles. When two and/or more pairs of large repeats are considered jointly, isometric master genome structures may generate many more subgenomic circles than those derived from the analysis of one pair of repeats. Thus, the subcircular molecular pool of the soybean genome is very large and complex, and includes 760 small circles of various sizes, which is more than the previous reports of less than tens of subgenomic mitochondrial circles in seed plants. Not all of the subcircles are listed here for simplification. The molecular pool is the most complex discovered among seed plants so far, which is directly related to the many pairwise large repeats in the soybean mitochondrial genome. Our results are qualitatively consistent with previous reports concerning classes of mitochondrial circles [18].

Whether or how the numerous subcircles generated by reversible homologous recombination affect mitochondrial function is unclear. The genes anchored in the subcircles were analyzed and found not to be clustered in subcircles according to their classes, such as respiratory Complex I-V, Cytochrome c, Ribosome, and *tRNAs*. In other words, the genes are dispersed throughout the subcircles, and the circles are not related to individual parts of the respiratory system. The dispersion of the genes among the mitochondrial subgenomic circles may enrich and diversify the molecular pool of the soybean mitochondrial genome. This may help to provide temporal control and generate sufficient transcripts to allow soybean to adapt to different internal or external environments.

### DNA Transfer between the Nucleus and Mitochondria

**Figure 5:**
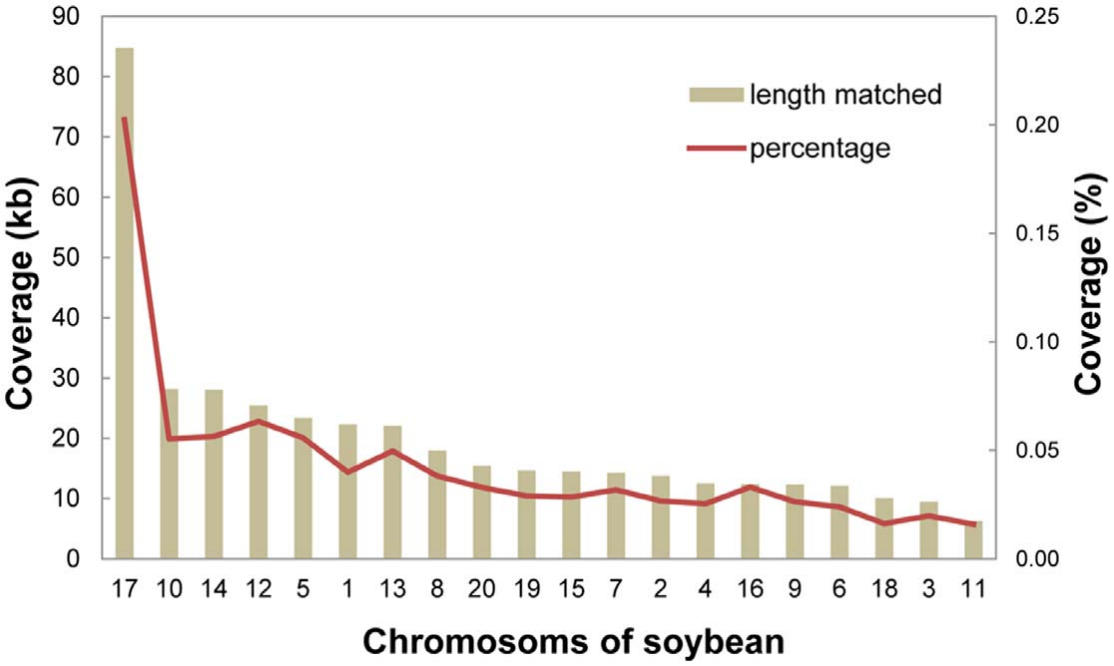
Chromosome coverage of hits obtained by searching the mitochondrial genome against the nuclear assembly. Rectangles show the lengths of matches covering the chromosomes. The lines show percent coverage by the matches on the soybean chromosomes.

The soybean mitochondrial genome was searched against the available soybean nuclear assembly genome [22] and the matches were filtered, which resulted in 1,866 hits covering 270.2 kb of the mitochondrial genome and 450.3 kb of the nuclear genome. The mitochondrial-nuclear alignment showed that hits occurred on every soybean chromosome (Figure 5); however, the total lengths of the hits and the percent coverage on the chromosomes are different. Chromosome 17 has the maximum total length of hits (84.8 kb) and the highest percent coverage (0.24%), much larger than other chromosomes. Chromosomes 18, 3 and 11 have the lowest coverage (<0.02%). Hits larger than 4 kb were identified on chromosomes. Chromosome 17 has four hits larger than 4 kb, chromosome 14 has two, and chromosomes 1, 8, 10, 12, and 13 have one hit larger than 4 kb. The largest hit is 9.5 kb in length on chromosome 17, and showed 98% identity. This region harbors exons D and E of *nad5*. Hits larger than 4 kb are all located on pericentromeric regions of the corresponding chromosomes (Table S6 in File S1). Thus, the pericentromeric regions are hotspots for DNA exchange between mitochondria and the nucleus. This transfer tendency is consistent with the chloroplast-nucleus transfer in rice [29].

**Figure 6:**
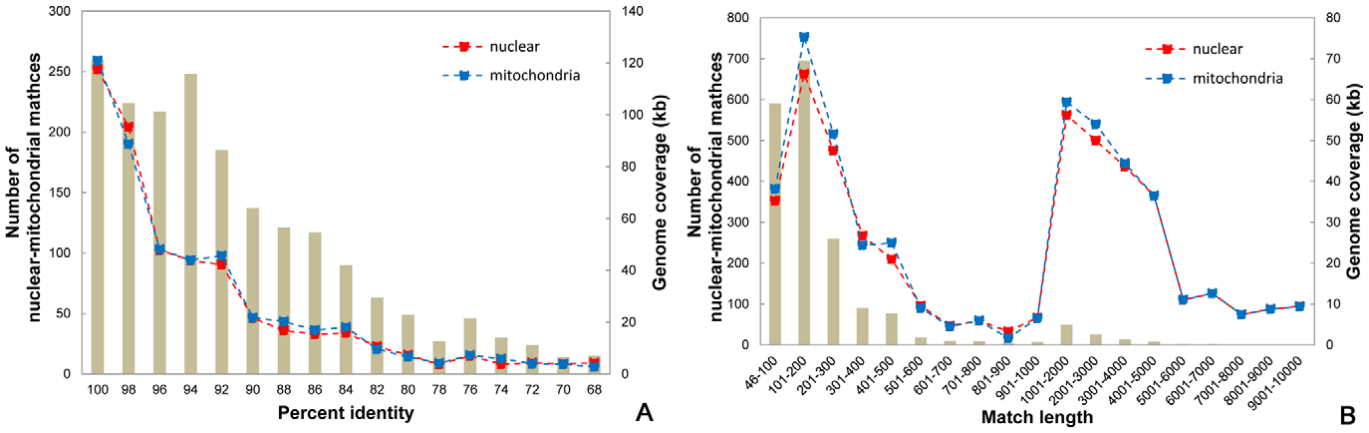
Characteristics of nuclear-mitochondrial sequences in soybean. Results are based on a BLAST e-value cutoff of 1e^–12^. (A) Distributions of percent identities between shared nuclear-mitochondrial matches. The number of matches is shown by brown boxes and is plotted on the left ordinate. The red and blue square lines show the coverage of matches on nuclear and mitochondrial genomes, respectively, and are plotted on the right ordinate. (B) Distributions of lengths between shared nuclear-mitochondrial matches; the notation method is the same as for (A).

The match length and percent identity of mitochondria-nucleus DNAs were jointly analyzed (Figure 6A). The number of shared nuclear-mitochondrial matches tends to sharply reduce as the percent identity decreases. A similar tendency is also found for the coverage length (Figure 6A). The number of shared nuclear-mitochondrial matches tends to decrease as the length of matches increases (figure 6B). This is probably caused by breaks and eliminations of long insertions in the two genomes, as inferred from the rice and *A. thaliana* genomes [30].

Sequences containing transposon sequences from the nuclear assembly may be identified as nuclear-derived DNA in mitochondria. These transfers in soybean account for 1.7% (6.8 kb) of the mitochondrial genome, and are located in 20 regions of the mitochondrial genome. Among them, one exogenous 2.1 kb DNA segment (the other segments are less than 1 kb) shows 94.8% identity to the nuclear transposon of RLC_Gmr24_Gm16–28 [31]. Regarding the types of transposons transferred into the mitochondrial genome, RLC, DTM and RLG (common nomenclature [32]) are found to have their vestiges at 13, 4 and 3 locations among the chromosomes, respectively. Thus, the RLC transposon is the major transposon that mediates sequence transfer to the mitochondria.

We identified 118 unambiguous nuclear copies of mitochondrial DNA (*numts*), based on their overlap with mitochondrial genes, introns, and pseudogenes. *Numts* account for 155.4 kb (0.02%) of the nuclear assembly. Most of these (91%) are <500 bp in length. The large *numts* with high percent identity to their cognate mitochondrial sequences suggest either a large number of recent transfer events or residual mitochondrial contaminants in the nuclear assembly. For the remaining ∼1400 matches, the direction of sequence transfer could not be determined; therefore they were treated as shared nuclear-mitochondrial sequences whose origin and inter-compartmental transfer direction are ambiguous [33]. These sequences cover more than one-third of the soybean mitochondrial genome (138.4 kb). No nuclear genes with complete ORFs were found to have transferred into mitochondria.

Almost all protein-coding genes of mitochondria have homologous regions in the nuclear genome of soybean, but only five mitochondrial genes (*cox3*, *atp4*, *nad4L*, *nad6* and *rps14*) have an intact ORF after transfer to the nucleus. The *numts* harboring the five mitochondrial genes are much longer than the ORFs of these genes (Table S7 in File S1). These gene transfers result from the direct transfer of bigger mtDNA sequences.

### DNA Transfer from Chloroplast to Mitochondria and Nucleus

With one exception, all DNA transfer between chloroplasts and mitochondria is unidirectional from the chloroplast to mitochondria [24]–[26], [28], [34], [35]. The exception is the transfer of a partial *cox1* gene sequence from mitochondria to chloroplasts in *Vitis vinifera* [36]. In our work, no mitochondria-specific conserved sequence was found in the soybean chloroplast genome [23], thus the unidirectional transfer principle may be applicable for soybean.

To identify the sequences transferred into the mitochondrial genome, the soybean mitochondrial genome sequence (without exons) was searched against the soybean chloroplast genome. Hits covering 3.9 kb (2.5%) of the chloroplast genome and 4.4 kb (1.1%) of the mitochondrial genome were identified. However, when the mitochondrial genome sequence is searched against angiosperm chloroplast genomes, the hits obtained cover 7.1 kb (1.8%) of the soybean mitochondrial genome. These results imply that loss of some sequences in the soybean chloroplast genome has occurred during evolution. The sequences lost from the chloroplast are maintained in the mitochondrial genome after ancient chloroplast-mitochondrial transfers. The sequences transferred to the soybean mitochondrial genome (*Mupts*) contain five chloroplast-characteristic complete genes (*trnM*-CAU, *trnH*-GUG, *trnN*-GUU, *trnD*-GUC, and *trnW*-CCA), and partial chloroplast gene sequences of *ycf2*, *psbH*, *rbcL*, and *atpI*. Among these chloroplast-derived genes, *trnM*-CAU and *trnH*-GUG do not exist in evolutionarily primary mitochondrial genomes of seed plants (Figure S4); however, the others have matches in the primary mitochondrial genomes.

To identify the nuclear DNA derived from the chloroplast genome (*nupts*), the chloroplast genome was searched against soybean nuclear assembly [22]. 7334 hits were obtained covering 151.6 kb of the chloroplast genome (99.6%). The *nupts* cover 1.1 Mb of the nuclear assembly (0.11%). Their coverage on the soybean chromosomes ranges from 35.3 kb on chromosome 11, to 69.1 kb on chromosome 18 (Figure S5). Except chromosome 17, which contains the maximum number of *numts*, the amount of DNA transferred from the chloroplast genome to nuclear chromosomes is 1.5-fold higher than that from the mitochondrial genome. Among the *nupts*, 24 chloroplast genes have complete ORFs, which account for 32.0% of chloroplast protein coding genes. Among the transferred chloroplast genes, 12 have been transferred as much larger cpDNA segments (Table S7 in File S1). The other 12 genes (*ndhC*, *rps4*, *psbM*, *petN*, *atpA*, *psaI*, *psbT*, *psbN*, *psbH*, *petD*, *ndhA*, and *psaC*) are located in *nupts* of almost equal length with the genes. The lengths of transferred DNA are associated with the mechanism for mediating the transfers, which will be discussed in the Discussion section.

### Horizontal Transfer

Two horizontal transfers in the soybean genome are found from bacterial genomes. One is a 106 bp segment homologous to *Staphylococcus simiae* CCM 7213, and the other is a 128 bp segment homologous to bacterial DNA (Table S8 in File S1).

The soybean mitochondrial genome has a 1.9 kb insertion sequence in *rps10* intron A. This insertion is also found in the mitochondrial genome of *V. radiate*, but does not exist in other mitochondrial genomes of seed plants. 0.5 kb of the 1.9 kb sequence is 57.4% identical to a mitovirus RNA polymerase gene, which might affect the function of the mitochondrial *rps10* gene. This is the first inserted virus sequence to be identified in the genic region of a plant mitochondrial genome. Furthermore, the other two legume mitochondrial genomes (*L. japonicas* and *M. pinnata*) do not contain this mitovirus sequence. Thus, the insertion probably occurred during recent evolution. The low identity to the reported virus sequences may result from a lack of genome information from the virus, or that the matched virus sequence is from a related virus. Three other insertion sequences by horizontal transfers are also homologous to the mitovirus RNA polymerase gene, ranging from 0.2 kb to 0.5 kb (Table S8 in File S1). Nevertheless, horizontal transfer from bacterial and virus species to higher plants is rare. They are probably the result of occasional events and seem to represent a major evolutionary component of the soybean mitochondrial genome.

### Comparative Analysis of Legume Mitochondrial Genomes

**Figure 7:**
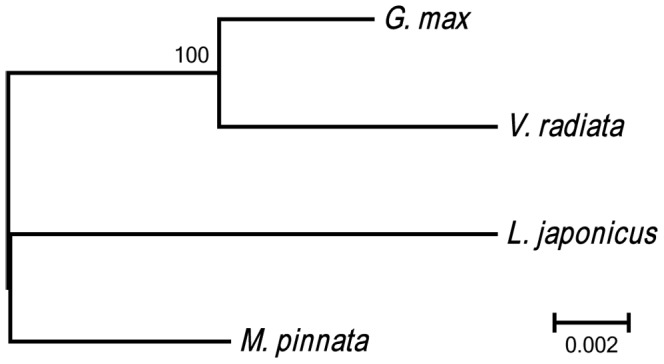
Phylogeny of four Faboideae mitochondrial genomes. Numbers above each node represent bootstrap values from 1000 replicates. Branch lengths are in units of synonymous substitutions per synonymous site.

The four legume mitochondrial genomes were clustered by neighbor-joining analysis based on synonymy substitutes (*d*_S_) of 30 protein-coding genes (Figure 7). The soybean genome is evolutionarily closest to that of *V. radiate*. The genomes of *M. pinnata* and *L. japonicas* belong to another class.

Alignment of the soybean mitochondrial genome with *V. radiate* produced 30 syntenic regions those were larger than 2 kb (Table S9 in File S1). This large number of syntenic regions indicates that the soybean mitochondrial genome may have experienced numerous rearrangements mediated by short repeats after diverging from *V. radiate*, leading to the significant structural disparity. Alignments among mitochondrial genomes from the four intergenus legume species consistently show large organizational disparities, indicative of the evolutionarily distant relationships with each other. On the other hand, the maximum length of syntenic regions produced from six comparisons among the four legume genomes is 13 kb. This syntenic region is not very big relative to those derived from closely related *Brassica* species [13], which implies that the four legume genomes have experienced many DNA reorganizations to form their present structures. The syntenic regions account for 44–63% of the mitochondrial genomes. However, these syntenic regions contain almost the same set of conserved coding genes of known function among the legume clade, indicating that the syntenic regions are maintained to harbor genes that are fundamental to the respiratory metabolism of legumes.

## Discussion

### Components of the Soybean Mitochondrial Genome

The size of the soybean mitochondrial genome is moderate relatively to the small *Brassica* mitochondrial genomes, which are mostly less than 300 kb [13], [17], [28]. The next largest mitochondrial genomes are those of cucumber and caryophyllaceae, which are usually more than 1 Mb [4], [33]. The determined size of the soybean mitochondrial genome is slightly different from the predicted size (392 kb), probably because of the different materials used.

The soybean mitochondrial genome contains sequences of multiple origins, including maternally-inherited coding gene sequences (18.2%) conserved in the syntenic regions, transfers from chloroplast DNA (1.8%), transfers from the nucleus (1.7%), and several horizontal DNA transfers from bacterial genomes and mitoviruses (0.3%) (Table 3). The coding genes of soybean account for a relatively high percentage compared with other higher plants, which range from 5.0% (*Vitis vinifera*) to 18.9% (*Citrullus lanatus*) [8], [37]. Soybean mitochondria have gained DNA from two other soybean genomes during evolution, which is consistent with those of other higher plants [7], [8].

**Table 3:** Features of the soybean mitochondrial genome.

	Feature	Nucleotides(bp)	Genome (%)
Coding	Protein exons	34,133	8.48
	*cis*-splicedintrons	32,553	8.09
	*rRNA*	5,276	1.31
	*tRNA*	1,427	0.35
Non-coding	Chloroplast-like	7,100	1.76
	Nuclear-like	6,809	1.69
	Mitochondrial-like	230,343	57.2
	Bacterial &mitovirus-like	1,045	0.26
Uncharacterized	Nuclear-shared	72,078	10.71
	Unknown	40,877	10.15

The coding sequences plus mitochondrial-like sequences identifiable in angiosperms account for 75.4% of the soybean genome. The sequences identifiable from ancient mitochondrial progenitors of the legume family account for about 71.8%, less than that identifiable in angiosperms. This is probably the result of significant deletions from progenitor genomes to form the specific mitochondrial genomes of the legume family. The sequences with uncharacterized origins account for more than 20% of the soybean genome, including sequences homologous to nuclear DNA (10.7%) and sequences of unknown origin (10.2%) (Table 3).

### Complex Structures in the Soybean Genome

The development of high-throughput sequencing has aided research into mitochondrial genomes. High sequencing depths for single copy genome segments and even higher depths for the repeats have helped to complete the connecting map, which is useful for analyzing mitochondrial genome structure. The mitochondrial genome of *Mimulus guttatus* was shown to comprise multiple circles by high-throughput sequencing [38]. We obtained the connecting map of the soybean mitochondrial genome using next generation sequencing technology, which demonstrated that it has numerous subgenomic circles and has the most complex genomic structures among the reported mitochondrial genomes of seed plants. The numerous circles coincide with the previously reported classes of subgenomic circles in the soybean mitochondrial genome [18]. However, no evidence of the existence of linear DNA or branched DNA, which were observed by electron microscopy, were noted [39]. Reasonable explanations for this include: (1) The soybean mitochondrial DNA looks like a network in the connecting map, making appearance of the branched DNA possible, and degraded mitochondrial DNA may represent the linear DNA observed by electron microscopy; (2) the previous study used different materials. Additionally, a mechanism for the replication of circular mtDNA has been proposed, but how the linear or branched DNA acts is unclear.

The large repeats could mediate the formation of multiple circular structures. This theory was demonstrated by experiments on restriction enzyme physical mappings and electron microscopy observations in *Brassica* and maize [11], [40], and has frequently applied to the analysis of mitochondrial genomes in higher plants [7], [12]. Based on this theory, the present work has predicted numerous subgenomic circles using pairs of repeats, based on the master circles. The results generated by this approach are consistent with the direct analysis of the connecting map. However, the analysis on the connecting map is difficult for many researchers; therefore, a theoretical method based on master circles combined with the connecting map is better than the two individual approaches.

The prediction of more than 700 subgenomic circles in the molecular pool of the soybean mitochondrial genome make the sequencing depths for contigs seemingly uniform, except for the repeats. What roles the circles play in the soybean life cycle remain unknown. However, the presented sequence represents a prerequisite to explore more deeply the roles of the circles.

### DNA Transfer

**Figure 8:**
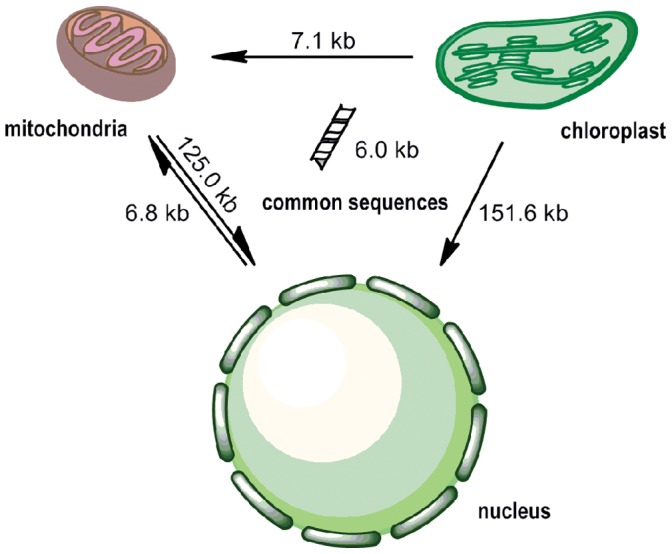
DNA transfers among the nuclear, chloroplast and mitochondrial genomes in soybean.

Our analysis of the intercellular DNA transfer in soybean is summarized in Figure 8: (1) The DNA segments transferred from the chloroplast and mitochondrial genomes into the nucleus are very large (>120 kb); (2) The transfer from the nucleus to the mitochondria is ambiguous because of the difficulty in determining the orientation of the transfers, except for small amounts of DNA sequences with vestiges of nuclear transposons (6.8 kb); (3) No DNA transfer was observed from the nucleus to the chloroplast and from the chloroplast to the mitochondria.

Some sequences are shared by the three soybean genomes. These common sequences represent 6.0 kb and include four complete genes (*trnD*-GUC, *trnH*-GUG, *trnN*-GUU, and *trnW*-CCA), and partial sequences of chloroplast genes (*atpA*, *atpI*, *ndhF*, *petG*, *psbH*, *rbcL*, *rps12*, *rrn16*, *rrn23*, *ycf2*, and *trnT*-UGU). The common sequences account for a very small proportion of the three genomes. Common sequences among the three soybean genomes may be regarded as transfers from the chloroplast.

The total length of DNA transfers varies with plant species. The total length of *nupts* ranges from 50 kb (*A. thaliana*) to 1.1 Mb (*O. sativa* subsp. *japonica*) in higher plants [41]. The *nupts* in soybean is 1.1 Mb,, which is a very high level. The total length of *numts* ranges from 71 kb (*Zea mays subsp. mays*) to 834 kb (*O. sativa* subsp. *japonica*) [41]; *numts* in soybean represent 155 kb, which lies in the middle of the range. *Mupts* in soybean represent 6.8 kb, which is moderate in comparison with the reported maximum in *Boea hygrometrica*, where about 40% of the chloroplast genome has been transferred to mitochondria [37], [42]. The *nupts* and *numts* contents have a strong positive correlation with nuclear genome size, and the abundance of *mupts* rises exponentially with mitochondrial genome size; thus, these transfers are influenced by the forces controlling the expansion and contraction of noncoding DNA of recipient genome [41]–[43]. The nuclear genome of soybean (∼950 Mb) had undergone whole genome duplication; thus, its genome size is towards the upper end of the scale in higher plants [22]. This may explain the high level of organelle sequence transfer into the soybean nuclear genome. The middle-ranking size of the soybean mitochondrial genome can explain the moderate number of *mupts*. The ratio of *nupts* and *numts* are also different among various species. The lowest is 0.09 in *A. thaliana* [41]. The ratio in soybean is 7, which is the largest in higher plants, probably because of the high ratio between the number of soybean mitochondria and chloroplasts within a soybean cell.

Horizontal transfer from viral and bacterial genomes to the soybean mitochondrial genome was detected for the first time in the present study. Further analysis indicated that the reads that constitute these DNA fragments are found across the mitochondrial conserved regions and the bacterial/viral homologous regions, which excluded the possibility they were contaminants. Although such transfers are rare in higher plants, the DNA transfer from mitovirus to mitochondrial genome always happens in the RNA polymerase region [7], [33], [36], [44], and the transfer probably influences the function of the mitochondria. Most transfers into mitochondria are recent events, restricted to closely-related species, such as a single plant genus [45]. The insertion into *rps10* of two legume species is consistent with these observations.

Physical mechanisms to explain the DNA transfer have been suggested by researchers. These include the lysis mechanism of organelles during autophagy, gametogenesis or fertilization [46], [47], a mechanism involving physical interactions between the nucleus and organelles [48], [49], a mechanism whereby abnormal mitochondria are taken up for degradation by the vacuole [50], the nuclear inclusion of mitochondria [51], and stromules connecting chloroplasts with mitochondria and/or the nucleus [52]. These mechanisms may provide conditions for the transfer of organelle DNA into nuclear genomes in soybean. Under the favorable conditions provided by the physical mechanism, organelle DNA may be integrated into the double-stranded breaks of nuclear genomes by non-homologous end-joining repair [53], [54]. The mechanism of mediation by the chloroplast stromules may also explain the DNA transfer from the chloroplast to the mitochondria in soybean.

The present study is the first to document DNA transfers between the mitochondria and the nucleus in both directions in soybean. DNA transfers from the mitochondria to the nucleus are less ambiguous than the reverse transfers, probably implying that transfer to the mitochondria is more difficult than to the nucleus. The mitochondrial genome is much smaller than the nuclear genome and the large number of mitochondria within a cell may help explain this difficulty. The smaller genome has fewer loci that can accept transfers; moreover, a mitochondrial genome that has accepted such transfers may not survive or may not be reproduced because of the negative effect on energy metabolism played by the transferred DNA.

Analysis of the match length between mitochondrial and nuclear DNA compared with the percent identity showed that as the length decreases, so do the corresponding percent identities. The mechanism suggested to explain chloroplast-nuclear DNA transfers in rice [29], may explain this phenomenon in soybean. DNA transfer may be regarded as a long-time dynamic evolutionary process in which the nuclear genome continuously integrates, shuffles and eliminates DNA from the mitochondrial genome. This results in the long insertions becoming smaller over time. However, this suggested mechanism for soybean is different from the conclusion derived from analysis of the cucumber mitochondrial genome [33] in which the mitochondrial genome has expanded by the absorption of much more DNA accompanied by less DNA elimination. Additionally, it was noted that the DNA transfers from the chloroplast to the nucleus in soybean show similar regulation to the chloroplast-nucleus transfers in rice (data not shown).

Several mitochondrial and chloroplast genes have been transferred to the nucleus in soybean. This is consistent with previous reports in higher plants such as *A. thaliana* and *O. sativa* [55]. The transfers of functional genes from an organelle to the nucleus are probably by direct transfer of organellar DNA sequences that are longer than the genic ORF. Genes with lengths equal to those of the ORFs themselves are probably created by reverse transcription of RNA [56]. Although large DNA fragments have been transferred from organelles to the nucleus in soybean, few of the transferred organelle gene sequences have retained their complete ORF in the nuclear genome of soybean. Even fewer genes transferred to the nucleus are functional, because only those genes that have acquired nuclear regulatory elements and have not been eliminated during evolution can be expressed [56]. In addition, four common tRNA genes among the three soybean genomes may be regarded as transfers from the chloroplast.

### Phylogenetic Analysis of Mitochondrial Genomes in Seed Plants

**Figure 9:**
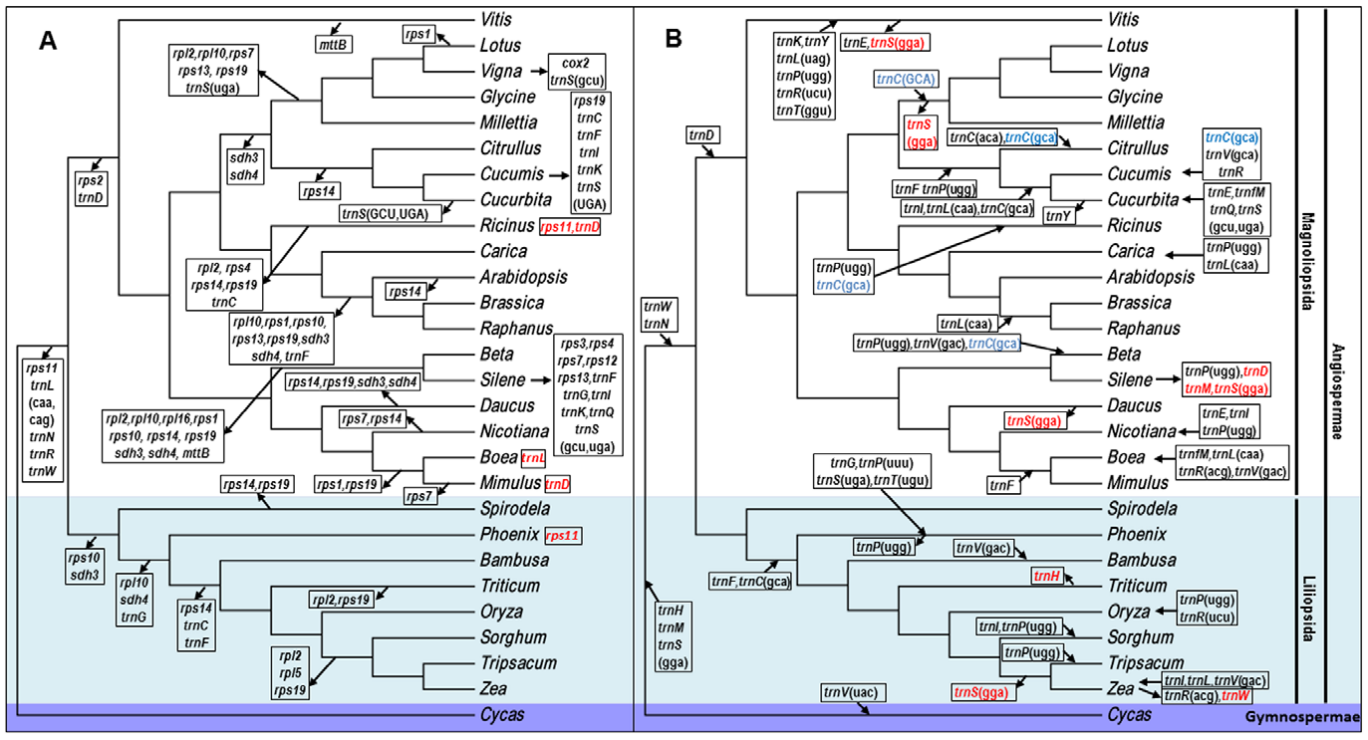
Phylogenetic tree of representative mitochondrial genomes in higher plants. Phylogenetic trees were constructed with 28 representative plant mitochondrial genomes using the coding sequences of 22 genes under the GTR+G+I model [66]. Mitochondrial-like tRNA genes and protein-coding genes eliminated during evolution are shown in (A) by arrowed black boxes in the evolutionary clades. Genes exceptionally maintained in the branches are shown by arrowed red boxes. (B) shows the chloroplast-derived tRNA genes transferred to the mitochondrial genome as boxes arrowed to a clade, and the genes lost in a branch are shown with an arrow to a box. *trnC* (GCA) represent tRNA genes of unknown origin, here attributed to chloroplast genes.

Recent progress has provided many more sequenced mitochondrial genomes. This provides unique insights into the phylogenetic relationships among mitochondrial genomes of plants. A phylogenetic tree with 22 conserved genes from mitochondrial genomes of 28 representative species of higher plants was constructed (Figure 9). The twenty-two conserved genes used in the analysis of mitochondrial genome evolution are *atp1*, *atp4*, *atp6*, *atp8*, *atp9*, *ccmB*, *ccmC*, *ccmFc*, *ccmFn*, *cob*, *cox1*, *cox3*, *matR*, *nad1*, *nad2*, *nad3*, *nad4*, *nad4L*, *nad5*, *nad6*, *nad7*, and *nad9*.

Evolutionary changes to mitochondrial genomes include not only mutations, but also losses of particular gene species and gene acquisition from the chloroplast genome. We labeled the gene changes in evolutionary clades and exceptional maintenance in some evolutionary branches to illustrate the evolution of the constituent genes in mitochondrial genomes of seed plants.

The number of protein-coding genes in mitochondrial genomes tend to decrease as plants become more evolved [57]. The genes lost are usually ribosome protein genes and succinate dehydrogenase genes. Few exceptional losses of genes include *cox2* in *V. radiate*, the protein product of which functions in the electron transport chain, and *mttB* in *Vitis vinifera,* which encodes a transport membrane protein. *Cycas taitungensis*, as a representative of gymnosperms, has conserved almost all of its mitochondrial genes that were inherited from the mitochondrial genome progenitor of seed plants [58]. *V. vinifera* has conserved the maximum number of mitochondrial genes in angiosperm plants, because it has lost only two genes (*rps2* and *rps11*) together with the transformation of the *mttB* gene into a pseudogene. *Silene latifolia* has lost the most genes, including all of the ribosome coding genes, except for *rpl5*, together with the *sdh* genes. Legumes, as angiosperm plants, show moderate levels of gene loss. In addition to the losses that characterize the evolutionary clades in which legumes are located, four legume plants (*V. radiate*, *G. max*, *L. japonicas* and *M. pinnata*) have lost *rpl10, sdh3* and *sdh4* genes (pseudogenes are regarded as lost genes).

The gradual loss of mitochondrial protein-coding genes has been observed in all clades of angiosperms (Figure 9A). Almost all of the Liliopsida and Magnoliopsida, except *Ricinus communis* and *Phoenix dactylifera*, have lost the *rps11* gene. This loss in the mitochondrial genomes probably occurred in the early stage of the divergence process between monocotyledons and dicotyledons. The loss of *rps2* probably occurred before the formation of Magnoliopsida plants. The loss of *rps10* is observed mainly in the differentiation process of Liliopsida. *rpl10*, *rps14*, *rps19*, *sdh3* and *sdh4* gradually developed into pseudogenes during the evolution of angiosperms. All ribosome protein genes can be considered unnecessary, to some extent, as indicated by their frequent absence in clades of angiosperm mitochondrial genomes.

Some mitochondrial-derived tRNA genes have also been lost during evolution. *trnL* (CAA and UAG), *trnN*, *trnR* and *trnW* genes were lost before the divergence of the Magnoliopsida from the Liliopsida. *trnD* genes do not appear in the Magnoliopsida; therefore, its loss must have occurred before the formation of the Liliopsida. The Liliidae have lost *trnC* and *trnF.* The legume family does not have *trnS* (UGA) (Figure 9A). Although many mitochondrial-like tRNA genes may be lost in various plants, the *trnE*, *trnP* and *trnY* genes are stably conserved in seed plant mitochondrial genomes.

In parallel with the loss of mitochondrial-like tRNAs, chloroplast tRNA genes have entered mitochondrial genomes through random intercellular transfers [34]. Some ancient transfers are maintained to some extent in many evolutionarily original species, and new transfers exist in individual species or genera. The *trnH*, *trnM* and *trnS* (GGA) genes from the chloroplast are frequently found in the mitochondrial genomes of *C. taitungensis* and the Angiospermae. These transfers may be considered to have occurred before the formation of angiosperms. Chloroplast-derived *trnW* and *trnN* are frequently found in angiosperms, *trnF* and *trnC* (GCA) are found in the Liliopsida, and *trnD* is frequently found in the Magnoliopsida. The presence of these genes in the different clades may have functional significance in seed plants. Chloroplast-derived tRNA genes are present sporadically in various clades and are probably absorption products of a late evolutionary stage. They may be functional in mitochondria, such as *trnS*-GGA, *trnF*-GAA and *trnC*-GCA of wheat mitochondria [59]. In addition, chloroplast-derived pseudogenes and loss of tRNAs may be observed. For example, *trnH* was inserted into mitochondrial genomes of seed plants, but disappeared in the *Triticum aestivum* mitochondrial genome. The *trnM* and *trnP* (UGG) genes in *Silene latifolia* turned into pseudogenes (Figure 9B).

The soybean mitochondrial genome has lost 16 genes, including nine protein-coding genes (*rpl2*, *rpl10*, *rps2*, *rps7*, *rps11*, *rps13*, *rps19*, *sdh3*, and *sdh4*) and seven tRNA genes (*trnS*(UGA), *trnW*(CCA), *trnR*(CCU), *trnN*(GUU), *trnL*(CAA), *trnL*(UAG), and *trnD*(GUC)) compared with the representative gymnosperm *Cycas taitungensis*, which has the maximum number of mitochondrial-like genes. The soybean mitochondrial genome has acquired five chloroplast-derived genes during its evolution. The 16 lost genes cannot be found in the other two soybean genomes. Thus, the genes lost may be unnecessary for soybean or their functions are duplicated by other genes. In other species, a few lost mitochondrial genes were found to have transferred into the nuclear genomes [60]–[62]; however, in general, the lost mitochondrial genes may be regarded as functional losses, or to have been redundant genes in seed plant genomes. This implies that gene loss may be regarded as the evolutionary compaction of the mitochondrial genome in seed plants.

## Materials and Methods

### MtDNA Isolation and Genome Sequencing

The soybean accession used was Aiganhuang (N21249), a typical landrace grown in the Huang-Huai river valleys of China, with similar Maturity Groups (III) to Williams 82 with sequenced nuclear genome. Mitochondrial DNA was extracted and purified according to the methods of Chen [17]. Genome sequencing was performed using the GS-FLX platform (Roche, CT, USA).

The sequences were assembled using Newbler v.2.6 (454 Life Science Corp, CT, USA) with default parameters. Each contig was aligned against the soybean nuclear and chloroplast genomes using BLASTn on soybase (http://soybase.org/), and searched against the nr/nt database in NCBI to remove the independent exogenous contigs. The fragments of other contigs that were homologous to the exogenous genomes (including the nuclear, chloroplast, bacterial and viral genomes) were positioned. Using the software Tablet [63] to open the ‘454contigs.ace’ file generated from Newbler, we searched the exogenous homologous fragments to determine the real transfer regions. Bb.454contignet software (http://www.vcru.wisc.edu/simonlab/sdata/software/) was used to visualize the contig connections of the *de novo* assembled sequencing data. Sanger sequencing was used to verify the contigs using a 3730×l (ABI, CA, USA). Lastly, we mapped the repeat regions and single copy regions of the soybean mitochondrial genome using Newbler and applied statistical analysis of the sequencing depth in these regions.

### Genome Annotation

The protein coding and rRNA genes were annotated using NCBI-BLASTn. The known mitochondrial genes of angiosperms were used as query sequences against our data. tRNAscan-SE (http://lowelab.ucsc.edu/tRNAscan-SE/) was used to annotate the tRNA genes. ORFs of more than 100 codons were predicted by ORF-Finder (http://www.ncbi.nlm.nih.gov/gorf/gorf.html). The circular map was drawn using OGDraw (http://ogdraw.mpimp-golm.mpg.de/).

### Genome Alignments

DNAs that had been transferred among the three soybean genomes (mitochondrial, chloroplast and nuclear) were identified by NCBI-BLASTn with stringent parameter settings. Soybean nuclear and chloroplast genomes used for alignment were from *G. max* PI 437654 (NC_007942) and *G. max* cv Williams 82, respectively, downloaded from plantdb (ftp://ftp.plantgdb.org/download/Genomes/GmGDB/). The e-value cutoff was set as 1e^−12^ when aligning mitochondrial and chloroplast genomes with the nuclear assembly and was set to 1e^−6^ when aligning mitochondrial with chloroplast genomes. Sequences on the nuclear scaffolds with coverage of more than 90% by the organelle sequences, and with identities of more than 90% to the organelle sequences were considered sequences polluted by organelle DNA, and were removed from the analysis. We used the soybase whole genome viewer (http://soybase.org/GlycineBlast Pages/) to analyze the distribution of hits on each nuclear chromosome of soybean.

The soybean mitochondrial genome was searched against the microbial genomes database (http://www. ncbi.nlm.nih.gov /sutils/genom_table.cgi) and mitovirus genomes (http://www.ncbi.nlm.nih.gov/genome/browse/) to detect the horizontally transferred sequences.

The soybean mitochondrial genome was searched against a database of representative seed plant chloroplast genomes using NCBI-BLASTn to detect conserved mitochondrial regions and identify chloroplast-derived sequences. All regions that did not match conserved mitochondrial regions or chloroplast-derived sequences were extracted and searched against transposable elements of soybean, which were downloaded from soybase (http://www.soybase.org/soytedb/#bulk) and the following databases in NCBI: the nucleotide collection database (nr/nt), the whole-genome shotgun contigs database (wgs), and the expressed sequence tags database (est).

In the genome alignments, the two sets of parameters used were the same as those used by Andrew [33], which are stringent setting (word_size 9, gapopen 5, gapextend 2, reward 2, penalty -3, dust no) and relaxed setting (word_size 7, gapopen 8, gapextend 6, reward 5, penalty -4, dust no). Stringent settings were used for the comparison between nuclear and organelle genomes and the relaxed settings were used for comparison between organelle and microbial genomes.

### Phylogenetic Analyses

The evolutionary tree of four legume species (*Glycine max*, *Lotus japonicus*, *Millettia pinnata*, *Vigna radiata*) was inferred using the Neighbor-Joining method for 30 genes. The bootstrap consensus tree inferred from 1000 replicates [2] was taken to represent the evolutionary history of the taxa analyzed. The evolutionary distances were computed using the Kumar method and are in the units of the number of synonymous substitutions per synonymous site. Evolutionary analyses were conducted in MEGA5 [64].

Twenty-eight species representing 28 plant genera were used to analyze the phylogenetic tree of the mitochondrial genomes. These species were *Arabidopsis thaliana* (NC_001284), *Bambusa oldhamii* (EU365401), *Beta vulgaris* subsp. *maritima* (NC_015099), *Boea hygrometrica* (NC_016741), *Brassica rapa* (NC_016125), *Carica papaya* (NC_012116), *Citrullus lanatus* (NC_014043), *Cucumis sativus* (NC_016005), *Cucurbita pepo* (NC_014050), *Cycas taitungensis* (NC_010303), *Daucus carota subsp. sativus* (NC_017855), *Glycine max* (JX463295), *Lotus japonicus* (NC_016743), *Millettia pinnata* (NC_016742), *Mimulus guttatus* (NC_018041), *Nicotiana tabacum* (NC_006581), *Oryza sativa Japonica* Group (NC_011033), *Phoenix dactylifera* (NC_016740), *Raphanus sativus* (JQ083668), *Ricinus communis* (NC_015141), *Silene latifolia* (NC_014487), *Sorghum bicolor* (NC_008360), *Spirodela polyrhiza* (NC_017840), *Tripsacum dactyloides* (NC_008362), *Triticum aestivum* (NC_007579), *Vigna radiata* (NC_015121), *Vitis vinifera* (NC_012119), and *Zea mays* subsp. *mays* (NC_007982). Exons of these genes were extracted and sequentially joined together. A Maximum Likelihood tree was constructed with MEGA 5 [64], using a general time reversible model. A discrete Gamma distribution was used to model the evolutionary rate differences among sites. The rate variation model allowed for some sites to be evolutionarily invariable. Codon positions included were 1st+2nd+3rd+Noncoding and the number of bootstrap replications was set as 1000.

## Supporting Information

Figure S1 **Agarose gel electrophoresis of PCR product in Table S1.**
(TIF)

Figure S2 **Connecting map.** Visualization of contig connections of the 454 sequencing assembled soybean mitochondrial genome, using bb.454contignet. The boxes contained mitochondrial genome contig ID and the depth of coverage assigned by Newbler. The numbers beside the contig lines show the depth of connection coverage. Numbers between arrows indicate number of reads common to both contig ends. The contig connecting order is as follows: 3-(15,R2)-12-24-27-8-(21-20-19-26-28,R1a)-1-(13-24,R3)-5-(16-28-26-19-20-23,R1b)-7-(24-13,R3) -6-(23-20-19-26,R1)-25-18-(17,R4)-11-(22-16-28-26-19,R1d)-9-(15,R2)-10-26-25-2-(22-16-28-26-19-20-21,R1e)-4-(17,R4)-14. The contigs in the parentheses showed the constitution of repeats.
(TIF)

Figure S3 **GC content distribution and sequencing depth distribution of the soybean mitochondrial genome.** Top image: GC content (/200 bp) of the soybean mitochondrial genome; Bottom image: sequencing depth distribution (/bp).
(TIF)

Figure S4 **Gene content in mitochondrial genomes of 28 representative species of higher plants.** Intact genes of mitochondrial origin are indicated by orange, pseudogenes as brown. Intact genes of chloroplast origin are indicated by green, pseudogenes as dark green. *trnC*(*gca*) of ambiguous origin are shown in blue. The missing genes are shown in white. This figure is modified from Andrew et al. (2011).
(TIF)

Figure S5 **Coverage of** ***nupts*** **on soybean chromosomes.** Rectangles show the length of matches covering the chromosomes. The lines show percent coverage by the matches on the soybean chromosomes.
(TIF)

File S1 **Additional tables.**
(PDF)
