# Correction: The Mitochondrial Genome of Soybean Reveals Complex Genome Structures and Gene Evolution at Intercellular and Phylogenetic Levels

**DOI:** 10.1371/annotation/5bf22546-6983-42c9-9cb5-1a6459b29a79

**Published:** 2013-06-27

**Authors:** Shengxin Chang, Yankun Wang, Jiangjie Lu, Junyi Gai, Jijie Li, Pu Chu, Rongzhan Guan, Tuanjie Zhao

Affiliations 1 and 2 for authors Shengxin Chang, Jangjie Lu, Junyi Gai, Rongzhan Guan and Tuanjie Zhao are incorrect. 

The correct affiliations are: 1 National Center for Soybean Improvement, Nanjing Agricultural University, Nanjing, China, 2 Key Laboratory of Biology and Genetic Improvement of Soybean, Ministry of Agriculture, Nanjing Agricultural University, Nanjing, China. 

