# Supplementary figures and images for: The Mitochondrial Genome of Soybean Reveals Complex Genome Structures and Gene Evolution at Intercellular and Phylogenetic Levels
Source: PLoS One. 2013 Feb 19;8(2):e56502. doi: 10.1371/journal.pone.0056502 (PMC3576410; doi:10.1371/journal.pone.0056502)

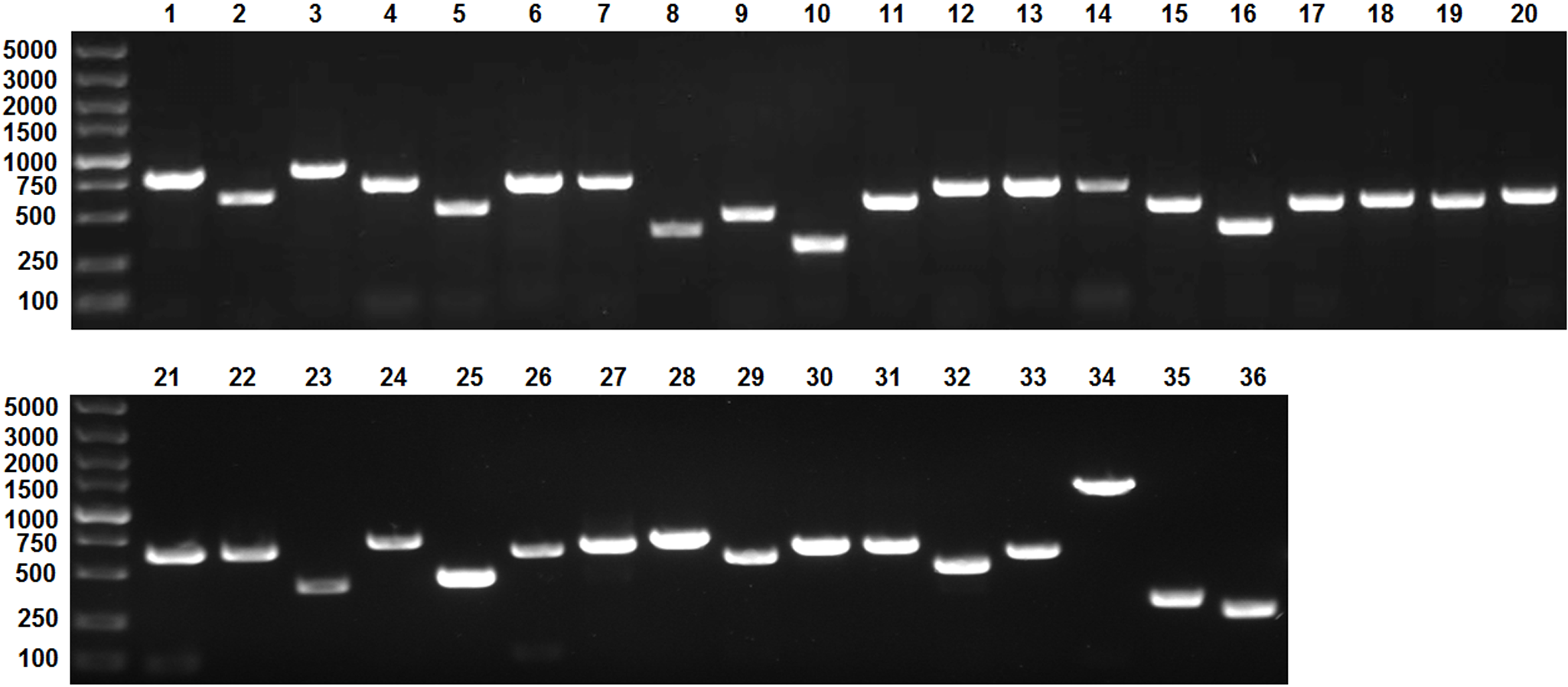

Supplement: Figure S1 — Agarose gel electrophoresis of PCR product in Table S1. (TIF) [file pone.0056502.s001.tif]

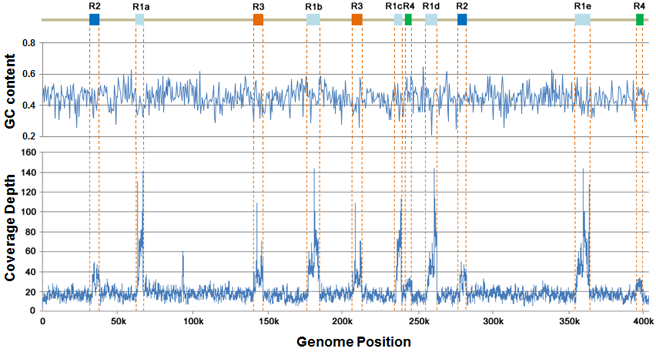

Supplement: Figure S3 — GC content distribution and sequencing depth distribution of the soybean mitochondrial genome. Top image: GC content (/200 bp) of the soybean mitochondrial genome; Bottom image: sequencing depth distribution (/bp). (TIF) [file pone.0056502.s003.tif]

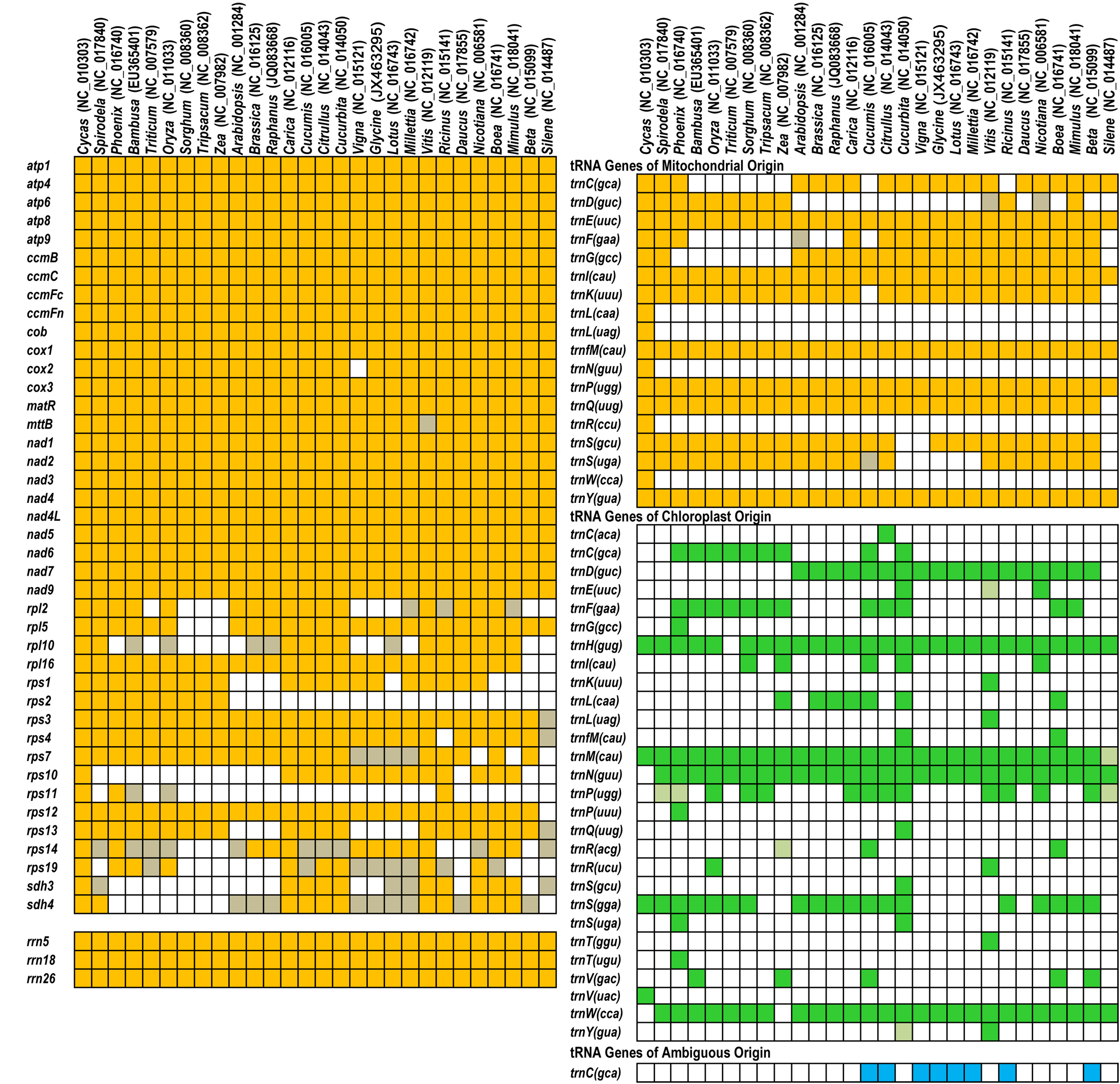

Supplement: Figure S4 — Gene content in mitochondrial genomes of 28 representative species of higher plants. Intact genes of mitochondrial origin are indicated by orange, pseudogenes as brown. Intact genes of chloroplast origin are indicated by green, pseudogenes as dark green. trnC(gca) of ambiguous origin are shown in blue. The missing genes are shown in white. This figure is modified from Andrew et al. (2011). (TIF) [file pone.0056502.s004.tif]

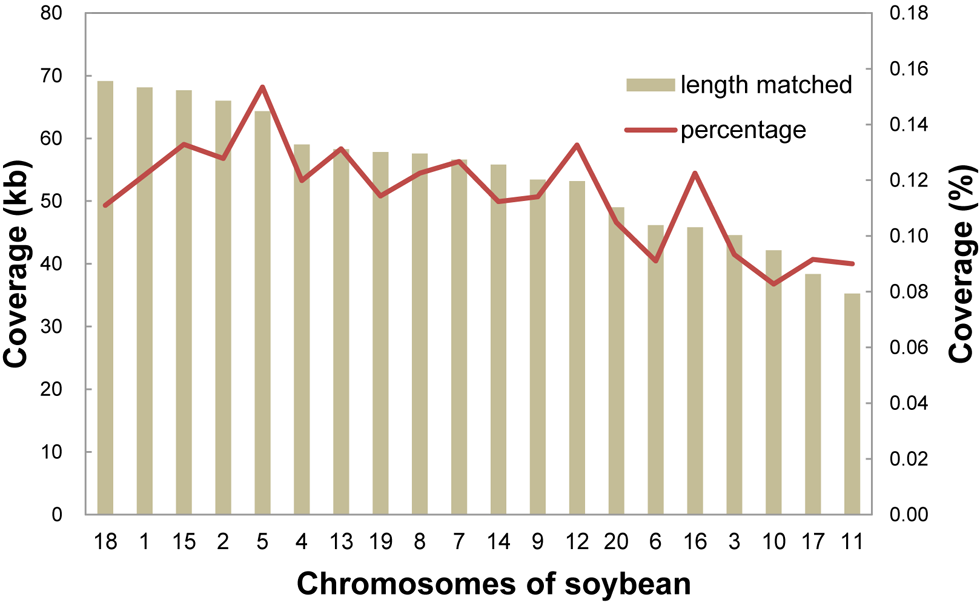

Supplement: Figure S5 — Coverage of nupts on soybean chromosomes. Rectangles show the length of matches covering the chromosomes. The lines show percent coverage by the matches on the soybean chromosomes. (TIF) [file pone.0056502.s005.tif]
