# Supplementary material for: The Mitochondrial Genome of Soybean Reveals Complex Genome Structures and Gene Evolution at Intercellular and Phylogenetic Levels
Source: PLoS One. 2013 Feb 19;8(2):e56502. doi: 10.1371/journal.pone.0056502 (PMC3576410; doi:10.1371/journal.pone.0056502)
Supplement: File S1 — Additional tables. (PDF) [file pone.0056502.s006.pdf]

Table S1. Primers for contig connecting verification

Name	Primer sequences	TM(℃)	Product length	Contig connections
1	F: 5'-TGCTCATCTGCTCTGGTT-3'<br>R: 5'-GTTTGACCAGTCCCAGCA-3'	54	763	contig1-13
2	F: CCTTTGTCTGGTAAGAAGTTGT-3'<br>R: 5'-CTTCCCTGTCTTGTCTTTCA-3'	53	684	contig1-28
3	R: 5'-CTTCCCTGTCTTGTCTTTCA-3'<br>F: 5'-CTCCTCCGTTACTCTCATCA-3'	54	835	contig2-22
4	F: CACCCTGGGAATTGGTTT-3'<br>R: 5'-ATCTGTTTCACCCCCGTT-3'	53	754	contig2-25
5	F: 5'-GCCAGGTTTGCTTATCCA-3'<br>R: 5'-TTCTGGCTGTCCTAACGA-3'	53	609	contig3-14
6	F: 5'-AGCAGTAGCGTAATACCACC-3'<br>R: 5'-GTGGACTGGCAACCTCTT-3'	52	702	contig3-15
7	F: 5'-CCATTACCTTATCCTTACCC-3'<br>R: 5'-GTGGCGTGGCTGAATGTA-3'	53	702	contig4-17
8	F: 5'-TCTTGGAAACGGGAGTGA-3'<br>R: 5'-CAAGGAGCAATCGTGAGG-3'	54	376	contig4-21
9	F: 5'-GCTTCTCTTTGCCCCTTA-3'<br>R: 5'-CTGCCCATTCACAAGGAC-3'	54	554	contig5-16
10	F: 5'-TGCTTGTTGAAGGGAGTG-3'<br>R: 5'-GATGCTTTTTGCTGCTATTC-3'	53	290	contig5-27
11	F: 5'-GTGGGAAAAGTCCGATTG-3'<br>R: 5'-ACCGATGGGTCTTGTTCT-3'	53	690	contig6-13
12	F: 5'-AGAAAGAAGGGGTCCGTT-3'<br>R: 5'-GCTGGGATAAGTACGGAA-3'	53	726	contig6-23
13	F: 5'-TCACTCTGGTGGAATCGC-3'<br>R: 5'-CTTTCCGAGACCAATGCT-3'	53	721	contig7-23
14	F: 5'-TCGCCGACTGCTACTAAG-3'<br>R: 5'-CCTGCCAACCAAGTCAAA-3'	53	710	contig7-24
15	F: 5'-CGGAACCCAAAGGCAA-3'<br>R: 5'-TCGTTTGCTAAGAAAGTGGA-3'	54	611	contig8-21
16	F: 5'-GGGAAGAAGTGGCATTTG-3'<br>R: 5'-GATGCTTTTTGCTGCTATTC-3'	53	389	contig8-27
17	F: 5'-CATCGGATTCCCTAAACA-3'<br>R: 5'-CTTTGAGTCGGCGATACA-3'	53	609	contig9-15
18	F: 5'-AAACAGGAGAAGGGACGA-3'<br>R: 5'-TCCCGAGAAAACGTGAAATA-3'	53	634	contig9-19
19	F: 5'-ACCCCCTATGACCGCTAT-3'<br>R: 5'-TGGTTATCCCCAAGGTTC-3'	53	547	contig10-15
20	F: 5'-AAGGCGGTTTTCTAAGTG-3'<br>R: 5'-TAGTCTCATTTTCCTTCGGC-3'	54	652	contig10-26
21	F: 5'-GATAGCATTTTGCGACCA-3'<br>R: 5'-TTCTAAAAAAGAGATGGTTGTG-3'	54	644	contig11-17
22	F: 5'-CTAAAAAGCCAAGGTCGC-3'<br>R: 5'-GCCCGAAAGAACACAAAG-3'	54	657	contig11-22
23	F: 5'-TTTATCTCGCTTGCCGTC-3'<br>R: 5'-TGGTTATCCCCAAGGTTC-3'	53	413	contig12-15
24	F: 5'-CCAAGGAAGCACTTACCG-3'<br>R: 5'-TCATTGGTTTCAACGGTG-3'	53	766	contig12-24
25	F: 5'-CAAGAACGATAAAGGCGA-3'<br>R: 5'-CCTGCCAACCAAGTCAAA-3'	53	458	contig13-24
26	F: 5'-ATAACTAAAGGTGCCAAGCC-3'<br>R: 5'-ATCTAAGTTCCCATCGGC-3'	52	602	contig14-17

27	F: 5'-ATAGAGTTGTTAGTTCCGCA-3'<br>R: 5'-CGAAAGGCACATAGAGGC-3'	52	678	contig16-22
28	F: 5'-GCATAGCCTTTCCCGC-3'<br>R: 5'-TGAAAGACAAGACAGGGAAG-3'	53	740	contig16-28
29	F: 5'-ATCTAAGTTCCCATCGGC-3'<br>R: 5'-GGACCCTGACTTACCTGACA-3'	53	576	contig17-18
30	F: 5'-TTTTTCATTTATGGTTGGGA-3'<br>R: 5'-ATCTGTTTCACCCCCGTT-3'	54	637	contig18-25
31	F: 5'-TGGCTTTTCGTTGAGGAC-3'<br>R: 5'-CTATGGCTCTACCAGGGAAT-3'	54	681	contig19-20
32	F: 5'-AAGACATCTATTTCACCCGT-3'<br>R: 5'-TAGTCTCATTTTCCTTCGGC-3'	55	572	contig19-26
33	F: 5'-AAAGAGAACCTGCCCTAAGA-3'<br>R: 5'-ACCCATTCAGACTCGCTT-3'	53	655	contig20-21
34	F: 5'-AATCCCTTACCAGCCGAG-3'<br>R: 5'-AGAAGCGATTCCACCAGA-3'	54	1451	contig20-23
35	F: 5'-TCATTGGTTTCAACGGTG-3'<br>R: 5'-GAATAGCAGCAAAAAGCATC-3'	53	351	contig24-27
36	F: 5'-AAGAGGTGGGAACGGG-3'<br>R: 5'-GAAGTGAAGTGAGCCTTACAAGAA-3'	58	209	contig25-26

contigsA-B means the connection of contigA and contigB. F denotes the forward primer, R denotes the reverse primer.

Table S2. Gene contents and total length of the gene sequences in the mtDNA of soybean

**Feature**	***G. max* (%)**
Total gene content	58
Protein-coding genes	36
rRNA	3
tRNA	19
Total gene length in bp	73,389 (18.23)
Protein exons	34,133 (8.48)
Protein introns	32,553 (8.09)
rRNA	5,276 (1.31)
tRNA	1,427 (0.35)

Data in parentheses are percentages that the total lengths account for in the genome.

Table S3. Frequency distribution of short repeats in the *G. max* mitochondrial genome

**Length**	**Number**	**Bases**	**Percentage (%) in the genome**
30-49	75	2,675	0.66
50-99	31	2,094	0.52
100-199	50	6,844	1.70
200-499	16	4,341	1.08
500-999	2	1,081	0.27
Total	174	17,035	4.23

Length column indicate the interval of repeat length. Bases mean the total length of short repeats in the interval. Percentage is that the short repeats account for the mitochondrial genome of *G. max*.

Table S4. Location and copy number of tandem repeats in the *G. max* mitochondrial genome

**Location**	**Period size**	**Copy number**
45,467-45,491	12	2.1
149,776-149,805	15	2.0
162,741-162,770	15	2.0
187,859-187,901	18	2.4

Indices indicate location of tandem repeats in the genome. Period size means unit length (bp) of tandem repeats. Copy number means the number of the repeats.

Table S5. Number of tandem repeats of seed plant mitochondrial genomes

	Species	Number	Accession	Reference
1	*Glycine max*	4	JX463295	Chang SX et al, 2012
2	*Boea hygrometrica*	5	NC_016741	Zhang T et al., 2011
3	*Lotus japonicus*	6	NC_016743	Kazakoff SH et al. 2012
4	*Millettia pinnata*	7	NC_016742	Kazakoff SH et al. 2012
5	*Nicotiana tabacum*	7	NC_006581	Sugiyama Y et al., 2005
6	*Vigna radiata*	11	NC_015121	Alverson AJ et al., 2011
7	*Spirodela polyrhiza*	12	NC_017840	Wang W et al., 2012
8	*Carica papaya*	14	NC_012116	Rice DW et al., 2011
9	*Citrullus lanatus*	14	NC_014043	Alverson,AJ et al., 2010
10	*Brassica rapa subsp. campestris*	17	NC_016125	Chang SX et al, 2011
11	*Brassica juncea*	17	NC_016123	Chang SX et al, 2011
12	*Brassica napus*	20	NC_008285	Handa H, 2003
13	*Oryza sativa Japonica Group*	20	NC_011033	Notsu Y et al., 2008
14	*Ricinus communis*	20	NC_015141	Rivarola M et al., 2012
15	*Brassica carinata*	20	NC_016120	Chang SX et al, 2011
15	*Raphanus sativus*	20	JQ083668	Chang SX et al, 2012
16	*Daucus carota subsp. sativus*	21	NC_017855	Iorizzo M et al., 2012
17	*Oryza sativa Indica Group*	22	NC_007886	Tian X et al., 2008
18	*Oryza rufipogon*	23	NC_013816	Fujii S et al., 2010
19	*Mimulus guttatus*	25	NC_018041	Mower JP et al., 2012
20	*Beta vulgaris subsp. vulgaris*	27	NC_002511	Kubo T et al., 2011
21	*Beta vulgaris subsp. maritima*	28	NC_015099	Darracq A et al., 2011
22	*Beta macrocarpa*	29	NC_015994	Darracq A et al., 2011
23	*Brassica oleracea*	33	NC_016118	Chang SX et al, 2011
24	*Triticum aestivum*	39	NC_007579	Ogihara Y et al., 2005
25	*Sorghum bicolor*	40	NC_008360	Allen JO et al., 2006
26	*Arabidopsis thaliana*	47	NC_001284	Unseld M et al., 1997
27	*Silene latifolia*	47	NC_014487	Sloan DB et al., 2010
28	*Phoenix dactylifera*	49	NC_016740	Fang Y et al. 2012
29	*Zea luxurians*	54	NC_008333	Allen JO et al. 2006
30	*Zea mays subsp. mays*	58	NC_007982	Clifton SW et al., 2004
31	*Zea mays subsp. parviglumis*	68	NC_008332	Allen JO et al., 2006
32	*Vitis vinifera*	72	NC_012119	Goremykin VV et al., 2008
33	*Zea perennis*	74	NC_008331	Allen JO et al., 2006
34	*Tripsacum dactyloides*	133	NC_008362	Allen JO et al., 2006
35	*Cycas taitungensis*	189	NC_010303	Chaw SM et al., 2008
36	*Cucurbita pepo*	281	NC_014050	Alverson AJ et al., 2010
37	*Cucumis sativus*	430	NC_016004-NC_016006	Alverson AJ et al., 2011

Table S6. Hits larger than 4 kb, obtained in search of the soybean mtDNA against nuclear assembly

Fragment	mtDNA range		Chromosome and range	Match length	Coding	Identity (%)	Pericentromeric
	Begin	End		(bp)	direction		region
1	8,578	13,084	Chr12:27,900,120..27,904,629	4,513	−	99.76	Y
2	45321	49834	Chr17:23,948,451..23,952,948	4,521	−	97.17	Y
3	101,825	110,599	Chr10:26,686,767..26,695,508	8,794	+	97.29	Y
4	104,798	111,184	Chr13:18,243,948 ..18,250,313	6,387	−	98.56	Y
5	120,226	125,140	Chr8:31,763,389..31,768,307	4,928	+	97.34	Y
6	129,421	133,893	Chr1:45,313,696..45,318,201	4,516	+	91.63	N
7	168492	173022	Chr14:10,573,916..10,578,379	4,550	+	90.2	Y
8	179041	183251	Chr17:23,999,916..24,004,135	4,231	−	97.64	Y
9	189159	196592	Chr17:23,978,091..23,985,529	7,441	−	98.9	Y
10	265667	275109	Chr17:23,959,619..23,969,014	9,464	−	98	Y
11	369643	375575	Chr14:10,567,993..10,573,917	5,950	−	94	Y

Table S7. The *numts*/*nupts* harboring integrity organelle genes

Fragment	Types	Location	Math length (bp)	Identity	Harboring Genes
1	*numts*	Chr12:27,900,120..27,904,629	4513	99.76	*cox3*
2	*numts*	Chr17:23,948,451..23,952,948	4521	97.17	*atp4*
3	*numts*	Chr17:23,971,313..23,972,533	1262	93.66	*nad4L*
4	*numts*	Chr8:41,260,207..41,263,604	3404	98.53	*nad6*
5	*numts*	Chr5:21,840,124..21,843,732	3665	94.98	*rps14*
6	*nupts*	Chr15:46,624,200..46,630,200	6118	96.68	*psbI*, *psbK*
7	*nupts*	Chr12:39,212,200..39,219,800	7640	97.34	(10)

10 genes on No. 7 fragment are *psbJ*, *psbL*, *psbF*, *psbE*, *petL*, *petG*, *psaJ*, *rpl33*, *rps18*, *rpl20*.

Table S8. Details of the soybean mitochondrial BLASTN matches to bacterial & mitovirus-derived sequences

Mitochondrial genome			Microbe genome		BLAST statistics	
Begin	End	Gene	Genbank ID	Description	Percent Identity	E-value
210,406	210,511	-	AEUN01000263.1	*Staphylococcus simiae* CCM 7213	99.06	2E-41
40,385	40,512	-	NC_008783.1	*Bartonella bacilliformis* KC583	70.31	2E-3
49,369	49,883	*rps10* intron A	NC_004053.1	*Ophiostoma* mitovirus 5 (RNA polymerase)	54.86	8E-4
89,072	89,549	-	NC_011372.1	*Botrytis cinerea* mitovirus 1 (RNA polymerase)	54.66	1.2E-2
127,357	127,663	-	NC_011372.1	*Botrytis cinerea* mitovirus 1 (RNA polymerase)	57.73	2E-5
160270	160445	-	NM_147269.5	*Arabidopsis thaliana* mitovirus (RNA polymerase)	66.67	9E-06

Table S9. Syntenic regions derived from alignment of the soybean mitochondrial genome with *V. radiate*

Code	*G. max*			*V. radiata*			Identities	Gaps	Strand
	**Begin**	**End**	**Length**	**Begin**	**End**	**Length**			
1	332003	345011	13009	56846	69809	12964	12778/13086 (98%)	199/13086 (2%)	Plus/Plus
2	93770	105257	11488	81605	92994	11390	11233/11553 (97%)	228/11553 (2%)	Plus/Minus
3	295103	305469	10367	123135	133514	10380	10190/10473 (97%)	199/10473 (2%)	Plus/Plus
4	77821	88035	10215	295379	305499	10121	9913/10327 (96%)	318/10327 (3%)	Plus/Plus
5	186040	195820	9781	148903	158593	9691	9543/9846 (97%)	220/9846 (2%)	Plus/Plus
6	159050	166248	7199	208247	215425	7179	7011/7248 (97%)	118/7248 (2%)	Plus/Minus
7	169804	176557	6754	343439	350172	6734	6632/6796 (98%)	104/6796 (2%)	Plus/Plus
8	57150	62861	5712	32488	38181	5694	5619/5736 (98%)	66/5736 (1%)	Plus/Plus
9	45159	50643	5485	395793	401262	5470	5334/5549 (96%)	143/5549 (3%)	Plus/Plus
10	317981	323437	5457	93700	99142	5443	5372/5477 (98%)	54/5477 (1%)	Plus/Minus
11	50644	55583	4940	1	4934	4934	4864/4972 (98%)	70/4972 (1%)	Plus/Plus
12	290030	294276	4247	224645	228845	4201	4108/4279 (96%)	110/4279 (3%)	Plus/Plus
13	251157	255155	3999	115409	119427	4019	3953/4031 (98%)	44/4031 (1%)	Plus/Minus
14	307757	311687	3931	6204	10147	3944	3884/3948 (98%)	21/3948 (1%)	Plus/Minus
15	243685	247589	3905	310753	314644	3892	3863/3909 (99%)	21/3909 (1%)	Plus/Minus
16	67593	71455	3863	239961	243789	3829	3750/3902 (96%)	112/3902 (3%)	Plus/Minus
17	37321	40961	3641	358625	362296	3672	3534/3726 (95%)	139/3726 (4%)	Plus/Plus
18	375461	379050	3590	132308	135188	2881	3400/3695 (92%)	214/3695 (6%)	Plus/Plus
19	336642	340227	3586	191386	194284	2899	2845/2915 (98%)	50/2915 (2%)	Plus/Plus
20	348819	352292	3474	364002	367477	3476	3404/3509 (97%)	68/3509 (2%)	Plus/Minus
21	328610	332017	3408	108231	111615	3385	3296/3455 (95%)	117/3455 (3%)	Plus/Minus
22	281406	284756	3351	273330	276706	3377	3278/3399 (96%)	70/3399 (2%)	Plus/Minus
23	19093	22393	3301	140819	144096	3278	3221/3321 (97%)	63/3321 (2%)	Plus/Minus
24	166235	169037	2803	277515	280303	2789	2745/2822 (97%)	52/2822 (2%)	Plus/Plus
25	266592	269261	2670	323150	325814	2665	2637/2674 (99%)	13/2674 (0%)	Plus/Minus
26	14450	16891	2442	205025	207471	2447	2390/2469 (97%)	49/2469 (2%)	Plus/Minus
27	108084	110485	2402	182696	185081	2386	2316/2434 (95%)	80/2434 (3%)	Plus/Minus
28	387274	389392	2119	384043	386152	2110	2066/2140 (97%)	51/2140 (2%)	Plus/Plus
29	181435	183508	2074	381826	383855	2030	2001/2082 (96%)	60/2082 (3%)	Plus/Minus
30	234781	236854	2074	381826	383855	2030	2001/2082 (96%)	60/2082 (3%)	Plus/Plus
